# Circulating microbiota and metabolites: Insights into cardiovascular diseases

**DOI:** 10.1002/jcla.24779

**Published:** 2022-11-29

**Authors:** Ikram Khan, Imran Khan, Muhammad Usman, Zhang Xiao Wei, Xie Ping, Sarmir Khan, Feroz Khan, Zhou Jianye, Li Zhiqiang, An Lizhe

**Affiliations:** ^1^ Department of Microbiology, School of Life Sciences Lanzhou University Lanzhou Gansu China; ^2^ School of Stomatology Northwest Minzu University Lanzhou Gansu China; ^3^ Department of Microbiology Khyber Medical University Peshawar Peshawar Khyber Pakhtunkhwa Pakistan; ^4^ State Key Laboratory of Grassland Agro‐ecosystem, Key Laboratory of Grassland Livestock Industry Innovation, Ministry of Agriculture and Ruler Affairs, Collegeof Pastoral Agriculture Sciences and Technology Lanzhou University Lanzhou Gansu China; ^5^ Department of Cardiology Lanzhou University Second Hospital Lanzhou Gansu China; ^6^ Department of Cardiology Gansu Provincial Hospital Lanzhou China; ^7^ Department of Reproductive Medicine, Academy of Medical Sciences The First Affiliated Hospital of Zheng University Zhengzhou Henan China; ^8^ Department of Zoology, Wildlife, and Fisheries PirMehr Ali Shah Arid Agriculture University Rawalpindi Pakistan

**Keywords:** bacteria, blood microbiota, cardiovascular diseases, circulating metabolites, non‐communicable diseases

## Abstract

**Background:**

In almost every country, cardiovascular diseases are the major cause of death, which are responsible for 17.7 million deaths worldwide, or 54% of all deaths. However, the latest evidence has shown that non‐communicable diseases such as obesity, diabetes, and cardiovascular events are significantly influenced by the blood microbiota and circulating metabolites.

**Methods:**

We searched online databases for the most recent related papers through the comprehensive international databases of the Institute of PubMed/ MEDLINE, ISI/WOS, and Scopus up to August 2022, using MESH terms and the related keywords in the English language. Considering the titles and abstracts, unrelated studies were excluded. The full texts of the remained studies were evaluated by authors, independently. Then, the studies' findings were assessed and reported.

**Results:**

The study demonstrated that the bacterial profiles of patients with cardiovascular diseases and healthy individuals are significantly different. The diseased patients showed a significantly high abundance of phylum Proteobacteria, an important Proteobacterial component known as lipopolysaccharides that has been linked to the pathogenesis of cardiovascular disease, while phylum Firmicutes were found in healthy individuals. It suggests that Proteobacteria has a direct role in the onset of cardiovascular disease.

**Conclusion:**

We focused on the blood bacterial composition and circulating microbial metabolites in their relationship with the etiology and onset of cardiovascular disease. However, the various genera and species in the results reported were not always identical. Therefore, the microbial community structure of blood was more complicated and thus required a more in‐depth exploration.

## Graphical abstract

Cardiovascular diseases are the major cause of morbidity and mortality worldwide. Several traditional risk factors and their associations with cardiovascular diseases has well known, however, the comprehensive study on circulating microbiota and metabolites in cardiovascular diseases has not been well explored. For the current study, we searched the relevant and latest articles using comprehensive international databases of the Institute of PubMed/ MEDLINE, ISI/WOS, and Scopus up to August 2022, using MESH terms and the related keywords in the English language. The irrelevant data were excluded at the time. The result of the study shows that the blood microbiota of patients with cardiovascular diseases is significantly distinct from that of healthy individuals.

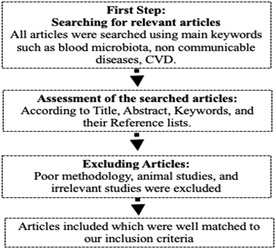

## 1 INTRODUCTION

Cardiovascular diseases (CVDs) are the leading cause of death worldwide.^1^ It is predicted that by 2030 the number of deaths due to CVD will increase to 23.3 million per year.^2^ For instance, a previous report has shown an estimated 17. 9 million people died due to CVDs in 2019, responsible for almost one‐third of all global deaths.^3^ Different risk factors such as age, gender, family history, hypertension, diabetes, obesity, smoking, and stress are involved in the attainment of the disease. Rapid changes in the environment and modern lifestyle (e.g., high consumption of meat, a diet rich in lipids, and physical activity) are contributors to underlie the increase in atherosclerosis and myocardial infarction (MI) worldwide.^4^ However, the meta‐analysis of prospective cohort studies reported no connotation between CVD and dietary saturated fat intake, signifying that other environmental factors are responsible.^5^ Moreover, the colonization of specific body sites in contact with the external environment (such as the gastrointestinal tract, skin, and vagina) by microorganisms is both well‐described and universally accepted,^6^ and the existence of microbial populations in other “classically sterile” locations, including the blood, is a relatively new concept.

The blood that flows perpetually through our veins and arteries performs numerous functions essential to our survival. Besides distributing oxygen, this vast circulatory system facilitates nutrient transport, deters infection, and dispenses heat throughout our bodies.^7^ Due to the long‐held belief that the bloodstream of healthy individuals is sterile and since the blood is an unfavorable compartment for the microbes due to its bacteriostatic and bactericidal components.^6^ Recent studies, particularly cross‐sectional studies targeting the 16 S rRNA gene, have revealed a dominant group of blood‐borne bacterial phyla (i.e., Proteobacteria, followed by Actinobacteria, Firmicutes, and Bacteroidetes),^8^, ^9^ and demonstrate the consistency of the human blood microbiota across time. Moreover, examination of the bacterial taxa reported in these studies reveals similar blood microbiota compositions across the different studies, whereby Proteobacteria dominate (relative abundance values typically ranging from 85% to 90%), and Firmicutes, Actinobacteria, and Bacteroidetes present to a lesser extent.^10^, ^11^, ^12^ This suggests the existence of a core blood microbiome profile that persists independent of the study environment or analytical methodology.

During the last 2 years, numerous studies have been published on blood microbial composition and its association with human health and diseases. For instance, several recent studies demonstrated a significant role of the tissue microbiome in the onset of cancer,^13^ other diseases,^14^ pre‐diabetic and type 2 diabetic patients,^15^ COVID‐19,^16^ metabolic diseases,^17^ portal hypertension,^18^ polycystic ovary syndrome,^19^ myocardial infarction,^20^ acute coronary syndrome, and chronic coronary syndrome.^21^ Moreover, microbial metabolites present in the gut include amino acid derivatives, hormones, short‐chain fatty acids (SCFAs), vitamins, and antioxidants.^22^ Such metabolites might be absorbed directly into the circulatory system of the patient. For instance, an association between endotoxemia (elevation of endotoxin from gram‐negative bacteria in the blood) and atherosclerosis in a population‐based study was discovered several decades ago.^9^ More recently, the deleterious effect of a bacterial metabolite, trimethyl amine oxide, on the blood vessel wall was shown in an animal model,^23^, ^24^ and the relevance of this finding in humans was suggested in a large prospective population‐based study.^25^

The current study aimed to summarize the intricate interplay between blood microbiota, circulating metabolites, and their putative roles in the development and progression of CVD.

## 2 CIRCULATING MICROBIOTA

**FIGURE 1:**
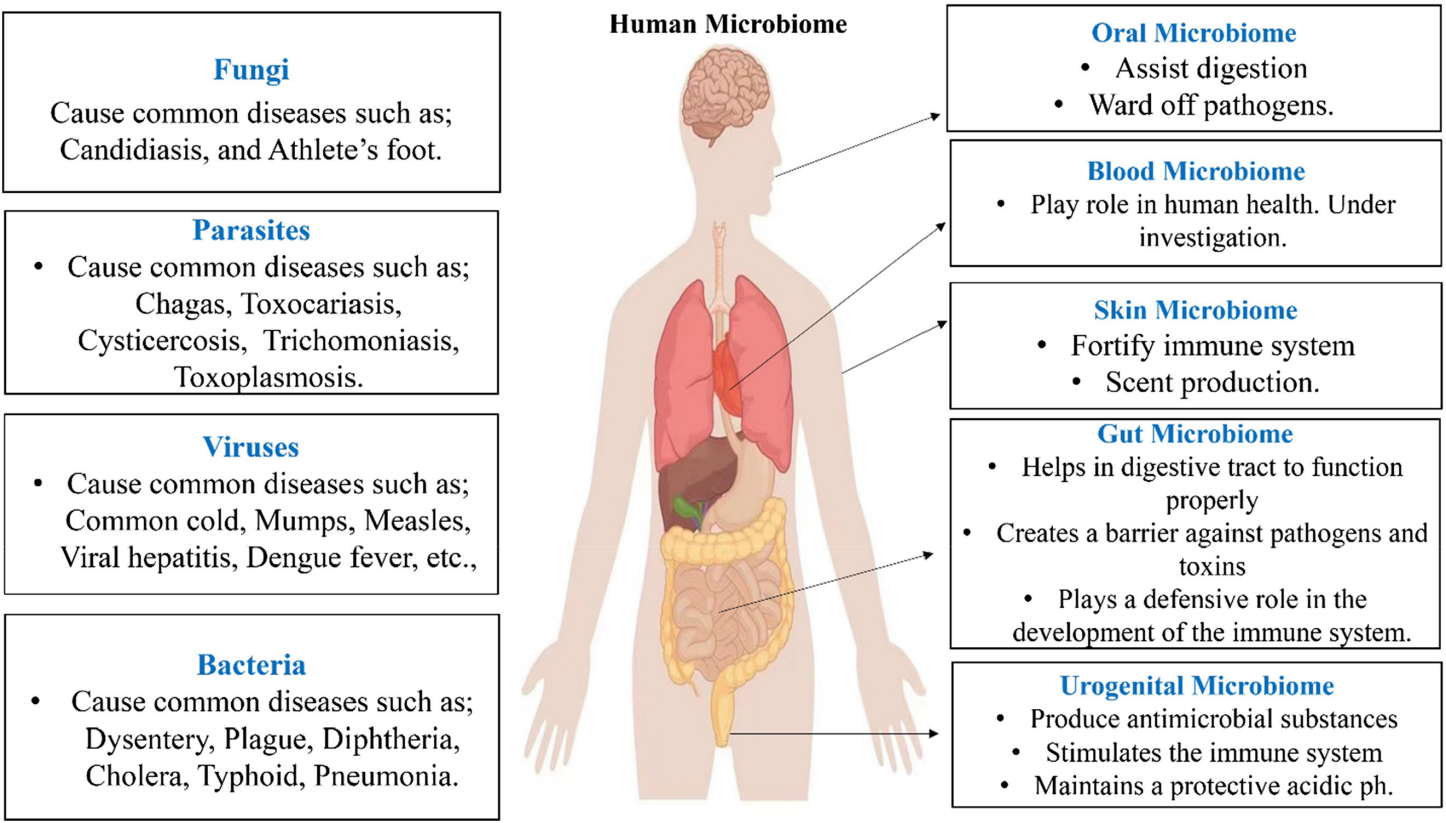
The human microbiome and its functions in the human body.

Different microbial communities exist within the digestive tract, on the skin surface, and nearly every visible surface of the human body is now well understood, as shown in Figure 1.^26^ A rising amount of studies suggests that increased permeability of oral and intestinal epithelial barriers may allow a limited number of microbes to reach the systemic circulation, then they can invade host organs and cause disease.^27^, ^28^ Bacteria that exist on or on exposed surfaces or that reach the systemic circulation can directly engage the innate immune system, prompting not only appropriate bactericidal responses but also altering host metabolism and inflammatory pathways connected to CVD.^29^ A high‐throughput 16 S rRNA gene sequencing has been used as a marker of microbial presence and a way of assessing bacterial diversity since the advent of next‐generation sequencing‐based technology.^30^ Two decades ago a study discovered bacterial 16 S rRNA in healthy human blood, the bacteria's final processes and presence had not been investigated.^31^ Although in recent years, a study used quantitative polymerase chain reaction to sequence 16 S rRNA gene V3‐V4 hypervariable regions in the blood of 30 young healthy volunteers to investigate the blood microbiome composition in the different fractions. The buffy coat had the highest concentration of bacterial DNA (93.74%), followed by RBCs (6.23%), and plasma (0.03%). In comparison to buffy coats and plasma, the red blood cell fraction had higher bacterial diversity.^11^ Unlike the gut taxa Firmicutes and Bacteroidetes, Proteobacteria were found in more than (80%) of blood samples, followed by Actinobacteria (10%) depending on the fraction.^11^ These results indicated that the blood microbiota is predominantly translocated from the gastrointestinal tract because most of the above‐mentioned bacteria at the phylum level have been discovered in the gut in previous investigations.^32^, ^33^ The skin and oral microbiota are more closely linked to the blood microbiota, with research on healthy blood suggesting that more bacterial translocation occurs from these niches rather than the stomach under normal physiology.^7^ Microbial DNA is often identified in cellular components in healthy people, and the microbial community composition of the healthy gut and blood showed significant alteration.^1^ In the gut, Firmicutes and Bacteroidetes phyla predominated, while Proteobacteria dominated in the blood.^11^ Healthy human circulating bacteria are considered dormant since it does not cause issues like sepsis and inflammation; yet, the circulating microbiome plays a crucial role in natural physiology and immunology.^34^ Furthermore, more research is needed to better understand the role of blood microbiota, physiology, and immunity in healthy individuals.

## 3 ALTERATION IN BLOOD MICROBIOTA WITH CARDIOVASCULAR DISEASES

The imbalance in host‐microbial interactions is known as microbiota dysbiosis. Enhanced disease susceptibility can be directly or indirectly linked to changes in microbial composition or community‐derived factors (such as metabolites or genotoxins). The pathophysiological diseases associated with CVDs may develop as a result of these microbiome‐based factors activating signaling pathways. Furthermore, it may be possible to target these components for therapeutic purposes (Table 1).

**TABLE 1:** Altered blood microbial compositions associated with CVDs

Disease and sample size	Sample	Technique	Disease‐associated changes in blood microbial abundance	References
70 ACS, 70 CCS, and 70 Controls	Whole blood	16 S rRNA sequencing	Actinobacteria phylum, and genus *staphylococcus* in the healthy group, phyla Proteobacteria, and Acidobacteria in the ACS group, and phylum Firmicutes, and genus *Lactobacillus* were found in the CCS group	21
29 MI patients and 29 controls	Whole blood	16 S rRNA sequencing	Actinobacteria phylum and *Bifidobacterium* genus were significantly increased in MI patients	20
20 RA patients	Serum	16 S rRNA sequencing	A high proportion of phylum Proteobacteria, and genus *Serratia*, *Corynebacterium* were observed in RA patients	53
103 non‐MI and 99 MI patients	Whole blood	16 S rRNA sequencing	In MI patients, the Caulobacterales order and Caulobacteraceae family were observed to be significantly lower	48
49 healthy, 100 STEMI, and 50 stable CAD patients	Blood leukocytes	16 S rRNA sequencing	*Streptococcus* spp., *Bacteroides*, and *Lactobacillus* were significantly higher in STEMI patients	43
727 incident stroke and 1312 incident CAD patients	Whole blood	16 S rRNA sequencing	Infections were connected to an elevated risk of CVD in both inpatient and outpatient settings	58
Portal hypertension 58 patients with liver Cirrhosis and 46 control patients	Blood	16 S rRNA sequencing	*Comamonas*, *Cnuella*, *Dialister*, *Escherichia/ Shigella*, and *Prevotella* were present in the patient group, whereas *Bradyrhizobium*, *Curvibacter*, *Diaphorobacter*, *Pseudarcicella*, and *Pseudomonas* numbers were decreased	18
48 VHD, 35 CHD, 50 IHD, and 45 controls	Whole blood	16 S rDNA analysis	All CVD patients had predominant *Staphylococcus* sp., while VHD patients have more bacteria detected than CHD and IHD patients	42
31 CVD patients and 10 controls	Whole blood	16 S rDNA sequencing	In CVD patients, there is a higher in *Pseudomonadaceae* and a lower in *Staphylococcaceae*, Gamma Proteobacteria, and Bacillales	41
80 CVD patients and 40 controls	Blood plasma	16 S rDNA sequencing	Actinobacteria predominated in CVD patients, whereas Proteobacteria predominated in healthy	40

**Abbreviations:** (ACS), Acute coronary syndrome; CCS, Chronic coronary syndrome; CKD, Chronic kidney disease; CAD, Coronary artery disease; (MI), Myocardial infarction; PD, Parkinson's disease; PCOS, Polycystic ovary syndrome patients; RA, Rheumatoid arthritis; (STEMI), ST‐segment elevation myocardial infarction; (T2DM), Type 2 diabetes mellitus.

Blood microorganisms were once thought to indicate infection.^21^ However, a rising body of evidence suggests that we should re‐evaluate blood sterility and embrace a normal microbiome in recent years.^35^ Thanks to technological advancements and the spread of novel techniques for analyzing the microbiota, such as 16 S rRNA sequencing and metagenome sequencing, recent studies have focused on the identification of bacteria that may be implicated in the genesis of endotoxemia and the development of metabolic disorders.^36^ The microbiome has an impact on human health and disease,^37^, ^38^ Although each microbial ecology differs, several species are ubiquitous.^38^, ^39^ Most of the studies that previously examined the relationship between the microbes and the onset of various diseases focused on the gut microbiota. However, researchers are now investigating links between blood microbiota and human health. The most prevalent bacterial phyla in the gut microbiota of healthy individuals are Proteobacteria, Bacteroidetes, and Firmicutes, while Proteobacteria predominate in the blood microbiota of healthy humans.^7^ Although previous studies found that CVD patients had higher levels of circulating microorganisms, with Proteobacteria and Pseudomonadaceae populations significantly higher and Firmicutes Gammaproteobacteria, Bacillales, and Staphylococcaceae populations much lower.^40^, ^41^ In addition, congenital heart disease (CHD), valvar heart disease (VHD), and ischemic heart disease (IHD) were all associated with elevated levels of *Staphylococcus* spp., in the blood circulation.^42^ The cardiovascular outcomes of individuals with ST‐segment elevation myocardial infarction (STEMI) are reportedly influenced by intestinal microbe translocation into the systemic circulation. These STEMI patients have elevated levels of *Lactobacillus*, *Bacteroides*, and *Streptococcus*, potentially due to gut barrier‐tight junction abnormalities.^43^ Previously the function of microbes in CVD was limited to Myocarditis, endocarditis, pericarditis, and rheumatic carditis are all caused by pathogen infection and species such as cytomegalovirus, *Chlamydia pneumoniae*, herpes simplex virus, *Porphyromonas gingivalis*, *Enterococcus* spp., *Streptococci* spp., *Helicobacter pylori*, and *Trypanosoma cruzi*, which are the main pathogens involved in these infections linked to heart diseases.^44^ The spread of these organisms via the bloodstream can cause bacteremia and sepsis, both of which can be fatal.^45^ These alterations in the circulating microorganisms may result in persistent inflammation and infection, which could cause CVDs.

Previously a study performed 16 S rRNA sequencing to analyze the microbial communities in whole blood from CVD patients and healthy controls, Proteobacteria were more abundant and Firmicutes were less abundant, but phylum‐level bacterial diversity remained the same. The same team found that Bacillales, Staphylococcaceae, and Gammaproteobacteria were significantly underrepresented in shotgun metagenome sequencing, but Pseudomonadaceae was significantly higher prevalent.^41^ Another study found that CVD patients had higher levels of proteobacteria/actinobacteria and circulating plasma microbial DNA. Additionally, the author discovered that although eukaryotic viruses were more prevalent in healthy adults, bacteriophages (*Pseudomonas*, *Rhizobium phages*, and *Propionibacterium*) predominated in the circulating virome of CVD patients.^42^ In addition, blood cultures from patients with Valvar heart disease, congenital heart disease, and ischemic heart disease showed an increased abundance of bacterial species particularly *Staphylococcus* sp.^40^ Pathogen infection was found to be a significant predictor of CVD risk 3 years ago in a study of 1312 incident coronary heart disease patients and 727 incident stroke patients.^45^ The most common cause of atherosclerotic plaques, which cause inflammation and CVD, is microbes in the bloodstream.^46^ A study discovered microbial translocation from the gut to the blood in individuals with ST‐segment elevation myocardial infarction and mice models by changing intestinal permeability, which is made up of tight junction proteins. As a result, gut bacteria such as *Streptococcus*, *Bacteroides*, and *Lactobacillus* were found in patients with ST‐segment elevation myocardial infarction.^47^

In the year 2019, Amar et al. found that cholesterol‐degrading bacteria (Aerococcaceae, *Rhodococcus*, *Gordonia*, Norcardiaceae, *Propionibacterium*, and *Chryseobacterium*) are present in the blood of control and MI patients, and the fractions of all of them are remarkably lower in MI patients. The Caulobacterales order and the Caulobacteraceae family were considerably lower in the MI group, and their presence in the blood of patients with myocardial infarction was likely to be negatively correlated with left ventricle ejection fraction.^9^ Recently our study also found that members of the phylum Bacteroidetes; class, Bacteroidia; and order Bacteroidales were significantly enriched in healthy controls (*p* = 0.05), while the phylum Actinobacteria; class, Actinobacteria; order, Bifidobacteriales; family, Bifidobacteriaceae; and genus, *Bifidobacterium* were significantly distinct in the myocardial infarction. Our study revealed a significant reduction in alpha diversity (Shannon index, *p* = 0.05), in the MI group when compared to the healthy controls.^20^ More recently, we performed another study that confirmed that the patients with myocardial infarction including (acute coronary syndrome patients, and chronic coronary syndrome patients) were distinct blood microbial profiles. Our study observed a significantly higher alpha diversity (Chao1, *p* = 0.001 and Shannon indices, *p* = 0.004) in the acute coronary syndrome group compared with the chronic coronary syndrome and healthy group, although a significantly lower alpha diversity was observed in the chronic coronary syndrome compared to acute coronary syndrome and healthy group, and beta diversity analysis demonstrated a major separation among three groups. Herein, we also found significantly distinct Proteobacteria in acute coronary syndrome patients, which suggests that the proteobacteria might play an important role in the onset of the coronary syndrome.^21^ Meanwhile, previously Amar et al reported that an increased Proteobacteria population in CVDs correlated with major factors like age, blood pressure, fibrinogen, and alanine aminotransferase levels.^48^ Also, recently several blood microbiota studies found an increased level of Proteobacteria in different chronic diseases. Thus, we conclude that Proteobacteria might play an important role in cardiometabolic diseases. Further studies are needed to investigate the direct role of Proteobacteria in the onset of cardiometabolic diseases.

Besides CVDs the blood microbiome may also have a role in the onset of other inflammatory diseases like Parkinson's disease and Alzheimer's disease.^34^, ^49^ Diabetes and cognitive diseases such as Alzheimer's disease and Parkinson's disease are linked. *Porphyromonas gingivalis*, the bacteria that cause chronic periodontitis, were detected in Alzheimer's disease patient's brain tissue, proving that the microorganisms circulated in the bloodstream from the oral cavity to the brain.^50^ Blood samples from individuals with Parkinson's disease, Alzheimer's disease, and type 2 diabetes mellitus were examined using correlative light‐electron microscopy since bacterial LPS impacts blood coagulation.^51^, ^52^ Since it has been suggested that the blood microbiota contributes to the pathophysiology of inflammatory illnesses, antibiotics, including antivirals, have been suggested as therapeutic agents.^49^ Another study investigated the circulating microbial DNA patterns of individuals with psoriatic arthritis, ankylosing spondylitis, and rheumatoid arthritis.^53^ In a different study that looked at cardiovascular death in patients with ESRD, 16 S and ITS rRNA could be found in all but three of the patients' serum samples. Even though there was no significant difference in 16 S rRNA levels of alpha diversity between cases and controls, the taxonomic analysis revealed different community profiles between groups, with significantly higher Actinobacteria and fewer Proteobacteria being observed at the phylum level in patients than controls.^54^ HIV also increased the number of circulating bacterial proteins. Additionally, HIV boosted the diversity of aerobic bacteria from the Micrococcaceae (Actinobacteria) and Pseudomonadaceae (Proteobacteria) genera, which predominated in the blood.^55^ Another study confirmed that the blood microbiome of rosacea patients had larger levels of the bacterial genera *Acidaminococcus* and *Megasphera*, as well as the genera *Rheinheimera*, and *Sphingobium* compared to the gut microbiome. Phylogenetic alpha diversity analysis by Faith revealed some differences between the blood microbiome of rosacea sufferers and controls.^56^

More recently, a study found that the relative abundance of Actinobacteria significantly increased whereas the relative abundance of Proteobacteria, Firmicutes, and Bacteroidetes significantly reduced in the polycystic ovary syndrome group. At various phylogenic stages, the cladogram showed the two populations' different microbiomes. The polycystic ovary syndrome group's Burkholderiaceae, Lachnospiraceae, Bacteroidaceae, Ruminococcaceae, and S24‐7 showed substantial declines in LDA, whereas Nocardioidaceae and Oxalobacteraceae showed significant increases. Furthermore, the findings demonstrated that the blood microbiomes of polycystic ovary syndrome patients had significantly lower alpha diversity, different beta diversity, and significant taxonomic alterations as compared to healthy controls.^19^ Additionally, the study found that the blood microbiota profile altered with age and that more than 95% of the blood microbiota belongs to the phylum Proteobacteria. The results of the clustering by principal component analysis showed clear patterns for each age group. In the elderly group (those over 60 years old), the class Gammaproteobacteria was found to be significantly higher, although the classes Alphaproteobacteria, Deltaproteobacteria, and Clostridia had significantly lower relative abundances (*p* = 0.05). According to alpha diversity measurements that considered the evenness of microbial taxa, the oldest group had significantly lower microbial taxa diversity than the other groups (Shannon index, *p* = 0.002 for young vs. elderly and middle vs. elderly, Dunn test), and slightly higher richness than the other groups (Chao1 index, *p* = 0.033 for middle verses elderly, Dunn test), there were no significant differences between the two groups.^57^ The influence of the gut microbiota on innate immunity and its significant impact on several non‐communicable diseases have been demonstrated and examined in recent studies. Consistently, the effect of oral microbiota in the pathogenesis of these diseases has been largely explored. Studies on blood microbiota provide some insights into the mechanism of action of circulating microbiota and serve as the first line of evidence for the involvement of tissue microbiota in these diseases. Various investigations, which were certainly observational or qualitative, were unable to establish the causality of or the importance of the blood microbiota in the genesis of these diseases. These investigations were also preliminary, and to confirm the existence of a stable blood microbiome in these disorders, the results obtained need to be repeated in a future study with a greater number of samples. Additionally, the bacterial DNA might operate as an uninvolved third party during the illness process. It is crucial to further understand the mechanisms governing the symbiosis of the blood microbiome and the molecular interactions between the host and bacteria.

## 4 CIRCULATING MICROBIAL METABOLITES

**FIGURE 2:**
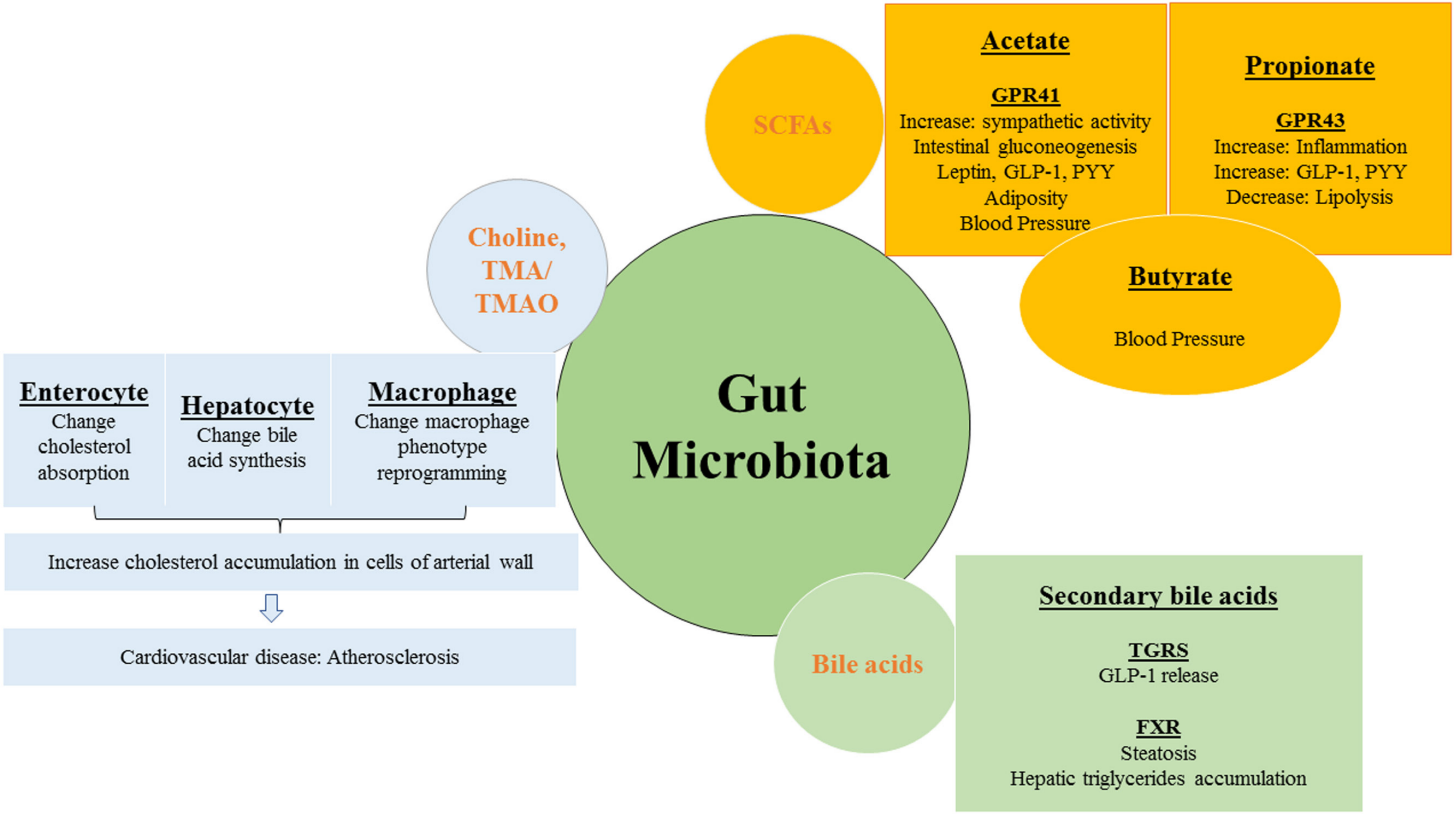
The mechanistic view of gut microbiota and circulating metabolites in the etiology and progression of CVD.

The diversity of functional genes in the microbiota demonstrates that the human microbiota has a higher metabolic capability than human cells, Figure 2. Dietary compounds and other substances are only partially digested by host systems; the remainder is processed by gut bacteria, especially in the anaerobic environment of the large intestine. The human microbiota also metabolizes medications, diverse synthetic compounds, and undigested carbs, lipids, and fiber, and can assess the efficacy or toxicity of these substances.^59^, ^60^ The gut microbiota should be considered when developing toxicological risk assessment methodologies for medications and environmental toxins.^61^ Our microbial cells metabolize a variety of compounds through proteolysis, reduction, hydrolysis, functional group removal, N‐oxide cleavage, denitration, deconjugation, amine formation, amide hydrolysis, thiazole ring‐opening, acetylation, and isoxazole scission. Various enzymes are involved, including azoreductases, esterases, lipases, nitroreductases, **β‐**glucuronidases, sulfatases, and **β‐**lyases.^60^

### 4.1 Trimethylamine N‐oxide

Trimethylamine N‐oxide (TMAO) has drawn a lot of attention among microbial metabolites as a significant cause of CVDs. Trimethylamine (TMA), which is predominantly produced through bacterial metabolism of dietary choline and phosphatidylcholine, produces TMAO as a byproduct in the liver.^23^ Studies on human cohorts and germ‐free mice have found a direct correlation between elevated CVD risk and plasma TMAO levels.^62^, ^63^, ^64^ Intestinal microbiota suppression or food supplement depletion can stop TMAO production and lessen atherosclerosis in mice fed choline‐ and carnitine‐rich diets, which resulted in high plasma TMAO levels and the development of atherosclerotic plaques.^23^, ^62^ Therefore, circulating TMAO may serve as a helpful CVD diagnostic marker. Increased risks of myocardial infarction may be indicated by elevated TMAO levels,^65^ heart failure,^66^ peripheral artery disease,^67^ stroke^68^ along with stable coronary artery disease,^67^ independent of the traditional cardiac risk factors.^69^ People who use broad‐spectrum antibiotics experience gut microbiota depletion and have large decreases in TMAO levels.^63^, ^69^ Additionally, people are advised to limit their intake of foods high in carnitine, choline, and lecithin since diet is a primary source of TMAO^62^ to lower the probability of developing a CVD. A previous study found that TMAO has been linked to modifications in plasma lipid, cholesterol, and sterol metabolism.^62^ Experimental evidence in animals has shown that TMAO affects vascular dysfunction, inflammatory reactions, and oxidative stress through contributing pathways.^70^ The TMAO receptor PERK in the endoplasmic reticulum has been found.^71^ It's interesting to note that TMAO has been shown to protect hemodialysis patients with vascular damage, maybe in part because of its inhibitory effects on AGE.^71^ It has been noted that several choline analogs lower TMAO levels in the blood. It has been discovered that the natural substance 3,3‐dimethyl‐1‐butanol, which is present in large quantities in red wines, vinegar, and various grape seed oils, inhibits the microbial choline TMA lyase activity. This substance could prevent the formation of atherosclerotic lesions in Apoe−/− mice without affecting blood cholesterol levels.^72^ There is evidence that several choline analogs, including fluromethylcholine, chloromethylcholine, bromomethylcholine, and iodomethylcholine, are more effective TMA lyase inhibitors that can lower plasma TMAO levels.^73^

### 4.2 Short‐chain fatty acids

Complex carbohydrates found in dietary fiber cannot be digested by the human intestine to support cell functions. However, fibers can be utilized by the gut bacteria through the fermentation process, which results in the generation of SCFAs.^74^ One to six carbon chains can be found in SCFAs, which are saturated fatty acids. Most SCFAs in the human body are found as acetate, propionate, and butyrate.^75^ SCFAs have a critical role in the regulation of gluconeogenesis, lipid metabolic pathways, and anti‐inflammatory responses. Additionally, these compounds, particularly butyrate, are thought to serve as energy sources for intestinal epithelial cells.^76^

The possible modulatory effects of the microbiota on CVDs are hypothesized to be mediated via SCFAs in systemic circulation. Numerous studies showed an inverse relationship between the operation of the prorenin receptor‐mediated intrarenal renin‐angiotensin system and the production of sodium butyrate and propionate by the gut microbiota, which helps to lower blood pressure.^77^, ^78^ Olfactory receptors 78 (Olfr78) and G protein‐coupled receptor 41(GPR41) are two examples of G protein‐coupled receptors that G protein‐coupled receptors (SCFAs) absorbed into the bloodstream may function through to mediate blood pressure effectors. Both receptors have distinct actions on vascular tone and are found in tiny resistance arteries. GPR41 behaves as a hypotensive protein to widen resistance arteries in an endothelium‐dependent manner when stimulated by SCFAs. By using Olfr78, this hypotensive effect can be countered.^79^ Under normal circumstances, these two receptors provide the required functional balance to stop excessive blood pressure volatility.^80^ Additionally, it has been shown that SCFAs generated from the microbiota have an immunomodulatory effect on reducing oxidative stress and maintaining a healthy immune system. It has been demonstrated that adding 1% butyrate to the diet slows the progression of atherosclerosis due to its anti‐inflammatory properties and improved plaque stability.^80^ In addition, T cell‐dependent protection from cardiac hypertrophy, fibrosis, vascular dysfunction, and hypertension is provided by SCFA propionate.^81^

### 4.3 Bile acids

The liver makes bile acid from cholesterol, which is then eliminated into the colon via the bile duct.^82^ Primary and secondary bile acids are two categories of bile acids.^83^ The two main bile acids in humans are cholic acid and chenodeoxycholic acid. They pass through the small intestine after being first excreted into the bile.^84^ Bacterial enzymes catabolize bile acids under the influence of bacteria, removing the hydroxyl group to create secondary bile acids such as ursodeoxycholic acid, deoxycholic acid, and lithocholic acid.^85^

Bile acids, however, have also been linked to an increased risk of CVDs.^86^ Adults with arrhythmias were more likely to have high taurocholic acid levels, and atrial fibrillation has also been linked to specifically bound bile acid concentrations, such as greater serum levels of non‐UDCA bile conjugates.^87^ Through FXR‐dependent FGFR4 signaling, bile acids may also lower plasma high‐density lipoprotein levels and liver paraoxonase‐1 expression.^88^ Oral metformin has been shown to influence gut microbiota, and bile acid metabolism, and block intestinal FXR signaling. Further investigation revealed that in type 2 diabetics receiving metformin treatment, the prevalence of Bacteroides fragilis was linked with modifications in bile acid metabolites and FXR signaling. Glycoursodeoxycholic acid (GUDCA) also exhibited therapeutic effects on insulin resistance and glucose intolerance.^89^ These results suggest that metformin improves metabolic dysfunction, including hyperglycemia, in part through a *Bacteroides fragilis*‐GUDCA‐intestinal FXR axis. In conclusion, the information at hand suggests that bile acids may contribute to the risk of CVDs, but more research is required.

### 4.4 Choline metabolites

Three phosphatidylcholine‐derived metabolites‐choline, TMAO, and betaine were found to be biomarkers for the prediction of CVD risk using untargeted metabolomic screening of a large clinical sample.^69^ A choline‐rich diet supplement increased the generation of TMAO and increased atherosclerosis in mice, showing that gut flora plays a role in CVD. After these choline metabolites were quantified, TMAO revealed a stronger relationship with an elevated risk of CVD, and taking antibiotics lowered the level of TMAO and prevented subsequent cardiovascular events.^90^ The primary microbial metabolite created by the metabolism of L‐carnitine, lecithin, choline, and betaine in the gut is trimethylamine (TMA). Flavin‐containing monooxygenase 3 is a liver enzyme that oxidizes TMA into TMAO in the liver. In population‐based and intervention studies, elevated plasma levels of TMAO have been linked to an increased risk of T2DM, cardiovascular and cerebrovascular diseases, incident thrombosis risk, carotid intima‐media thickness, and STEMI.^91^ Recently, a study found that individuals with heart failure had elevated levels of TMAO and indoxyl sulfate and that these levels were related to the prevalence of Escherichia and Shigella spp. in the gut microbiota.^90^ To establish TMAO as a therapeutic target in CVD, more studies are needed to elucidate the precise mechanism of action of TMAO on vascular complications.

### 4.5 Other metabolites

Aromatic amino acids like phenylalanine, tryptophan, and tyrosine might affect immunological, metabolic, and neural responses. Tyrosine, which can be further converted into neurotransmitters, norepinephrine, and adrenaline, is a metabolic precursor for phenylalanine, an important amino acid. Tryptophan is an important amino acid that serves as a precursor to another neurotransmitter, serotonin. A microbiota derivative of tryptophan was dramatically reduced in plasma in people with advanced atherosclerosis.^92^ Tyrosine and phenylalanine metabolites in the intestinal microbes have been associated with MI severity in rats, according to a recent study.^93^ There needs to be more research into the molecular linkages between these aromatic amino acid metabolites and cardiovascular disorders. The amino acid phenylacetylglutamine is formed when dietary phenylalanine is metabolized to phenylacetic acid and has been linked to CVD, and significant hostile cardiovascular events.^94^ Signaling through adrenergic receptors, this gut microbiota‐derived chemical enhances platelet activation‐related characteristics and thrombosis potential.^95^

## 5 MICROBIOTA‐TARGETED THERAPEUTICS

**FIGURE 3:**
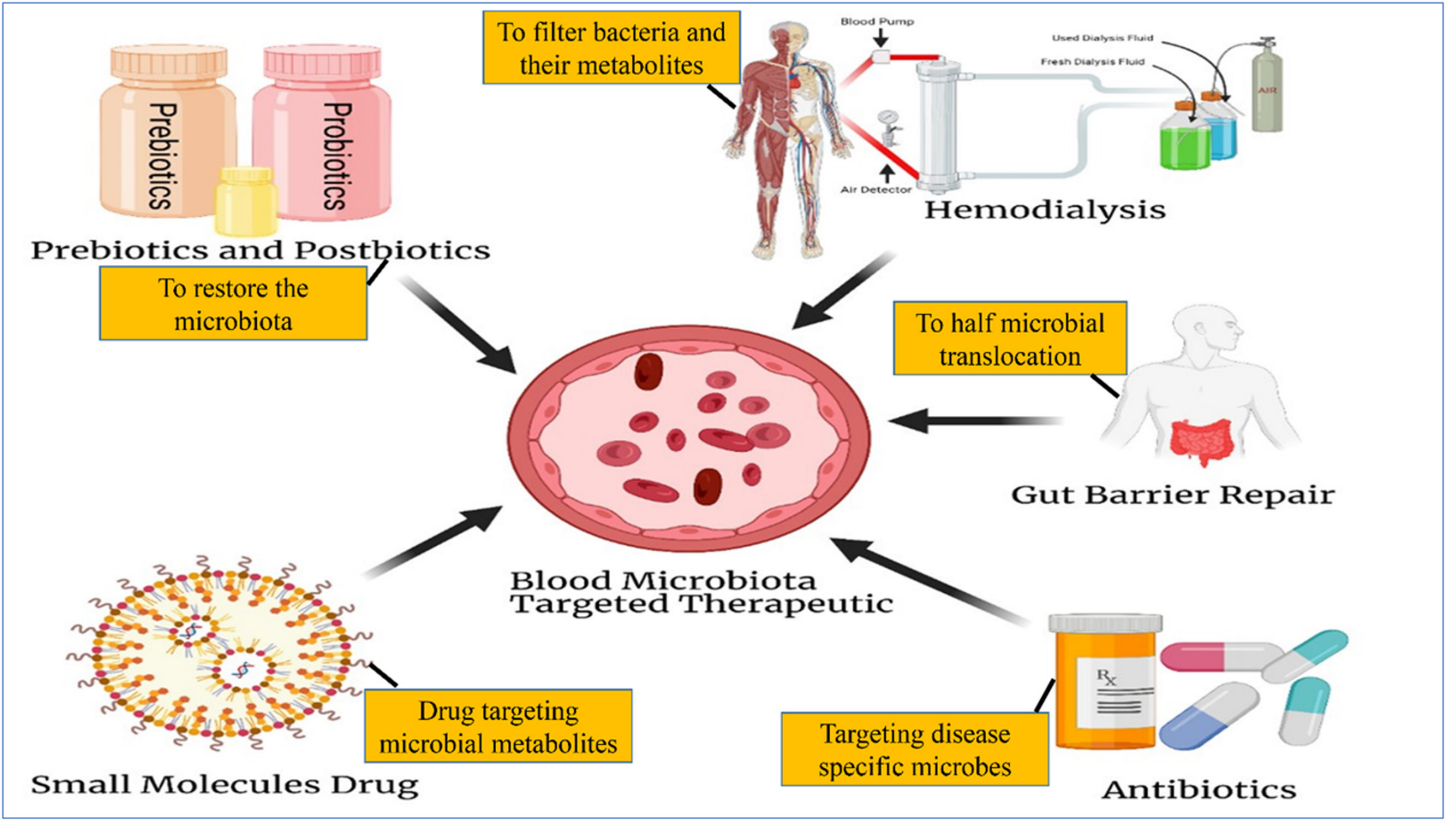
Treatment strategies for non‐communicable diseases that focus on the blood microbiome.

Modern techniques are being utilized to identify changes in the blood microbiota and circulating metabolites, including metabolomics and next‐generation sequencing. The advancement of biosensors and nano‐sensors for the detection of specific bacteria or their metabolites will facilitate the incorporation of these biomarkers into routine clinical analysis. The restoration of the blood microbiota and circulating metabolites has been accomplished using a variety of techniques as an alternative therapeutic approach, Figure 3. To repair the intestinal barrier and restore the balance of the blood microbiota, prebiotics, probiotics, and antibiotics have all been used,^23^, ^69^, ^96^, ^97^, ^98^, ^99^ restoring the diversity of the bacteria in the gut, which may have a big impact on the blood microbiota and its circulating metabolites. Hemodialysis, a treatment for kidney failure that is often used, may be a therapeutic strategy to help diseased people's blood microbiotas repair. Hemodialysis membranes can be made to precisely filter intestinal microbes and their derivatives. For example, utilizing the oral charcoal adsorbent, indoxyl sulfate elimination from patients with advanced renal failure has been clinically performed.^26^ Small synthetic chemical compounds are being developed into drugs that target the microbiota, their metabolites, and host mediators; some of these are currently in clinical trials.^99^, ^100^

## 6 FUTURE PERSPECTIVES AND DIRECTION

Good scientific practice is important in all areas of science. In recent years, this has gained more and more attention, especially considering the scientific reproducibility crises.^101^ While most researchers are aware of the issues with good scientific practice, not all these issues are necessarily clear, and the details can be very complicated. For many years, it has been accepted to perform and publish sequencing‐based microbiome studies without including proper controls. Although in recent years more scientists realize the necessity of implementing controls, this poses a problem due to the complexity of the field. Another concern is the inability to properly interpret the information gained from controls in microbiome studies. In the microbiological sector, contamination is one of the main problems with 16 S‐based categorization, especially when extremely small amounts of bacteria and compounds produced from bacteria are present.^101^, ^102^ The presence of environmental pollutants, laboratory reagents, and individuals participating in sample preparation in this situation could have a significant impact on the findings of the investigations and result in incorrect conclusions. It is noteworthy that longitudinal investigations like the one from Turner et al. have demonstrated the significance of the timing of nucleic acid extraction,^103^ due to the possibility that various batches of the same extraction kits could identify different contaminants.^104^ These illustrations unmistakably show that to account for contaminants and create solid and trustworthy results, microbiological investigations should always contain several negative controls.

It has now become evident that highly diverse microbial communities exist in the systemic circulation of various populations and that both quantitative and compositional changes in the circulating microbiota may contribute to the development and progression of cardiometabolic disease. However, many important questions remain unanswered regarding the nature of circulating microbiota, including the sources, pathophysiological roles, localization across different blood fractions, and distinction from potential contamination, all of which need to be clarified in future in‐depth basic, clinical, and population‐based research. In an ongoing quest to improve outcomes of patients with cardiometabolic disease, perhaps the time has come to go beyond the “gut feeling” and rigorously incorporate the potential pathophysiological insights gained from the circulating microbiota toward the development of novel biomarkers for diagnosis and prognosis, especially in personalized therapeutic approaches to premature morbidity and mortality in cardiometabolic disease where patterns are emerging.

## 7 CONCLUSIONS

In this review, we have selected a few of the most recent cross‐sectional studies of human cohorts that have made it possible to characterize the alterations in the blood microbiome linked to CVDs and other diseases. The ecological differences (dysbiosis), biological entities (“new species”), and gene‐based variations (“biomarkers”) related to illness diagnosis and the effects of current treatment therapies on the circulating microbiota have all been the subject of new insights from these investigations. Numerous studies have firmly established the significance of the blood microbiome in the emergence of cardiovascular and other disorders. The “omics”‐based techniques are offering new insights into disease pathogenesis despite the complexity of the microbiome and the confounding effects resulting from host genetics, nutrition, medical co‐morbidities, and other lifestyle factors. Certain could result in the creation of therapies that could be used to treat and prevent these metabolic illnesses. It is challenging to properly grasp these microbes’ involvement in disease development and prevention and to use this information to apply microbiome research to medicine, as many of these microbes are still difficult to cultivate. To effectively complement and communicate the advancements being made by microbial genomics, there is an urgent need for a resurgence of interest in microbial biology. The ability to provide individualized therapeutic and/or preventative interventions, such as “next generation,” logically assembled microbial consortia and probiotics, as well as dietary changes for reestablishing a gut microbiota that promotes and sustains health, will be supported by bringing metagenomic data to life, in the form of viable microbes.

## AUTHOR CONTRIBUTIONS

Supervised: An Lizhe and Li Zhiqiang; concept and write‐up: Ikram Khan; revised: Zhou Jianye, Zhang Xiao Wei, and Xie Ping; figures preparation: M. Usman, Imran khan, Ikram Ali, and Sarmir Khan; and funding acquisition: An Lizhe and Li Zhiqiang.

## FUNDING INFORMATION

This work was supported by the (31760159).

## CONSENT FOR PUBLICATION

All the authors agree to publish this article.

## Data Availability

All processed data used in this study are included in the article.

## References

[1] Khan I , Khan I , Jianye Z , et al. Exploring blood microbial communities and their influence on human cardiovascular disease. J Clin Lab Anal. 2022;36:e24354. doi:10.1002/jcla.24354 35293034 PMC8993628

[2] Midkhatovna SE . Efficacy and safety of lipid‐lowering drugs as primary and secondary prevention of cardiovascular diseases in the elderlyin the uzbekistan. Int J Cult Mod. 2022;13:68‐75.

[3] Roth GA , Mensah GA , Johnson CO , et al. Global burden of cardiovascular diseases and risk factors, 1990–2019: update from the GBD 2019 study. J Am Coll Cardiol. 2020;76(25):2982‐3021. 33309175 10.1016/j.jacc.2020.11.010 PMC7755038

[4] Zununi Vahed S , Barzegari A , Zuluaga M , Letourneur D , Pavon‐Djavid G . Myocardial infarction and gut microbiota: an incidental connection. Pharmacol Res. 2018;129:308‐317. doi:10.1016/j.phrs.2017.11.008 29133215

[5] Siri‐Tarino PW , Sun Q , Hu FB , Krauss RM . Meta‐analysis of prospective cohort studies evaluating the association of saturated fat with cardiovascular disease. Am J Clin Nutr. 2010;91(3):535‐546. 20071648 10.3945/ajcn.2009.27725 PMC2824152

[6] Whittle E , Leonard MO , Harrison R , Gant TW , Tonge DP . Multi‐method characterization of the human circulating microbiome. Front Microbiol. 2019;9:3266. doi:10.3389/fmicb.2018.03266 30705670 PMC6345098

[7] Castillo DJ , Rifkin RF , Cowan DA , Potgieter M . The healthy human blood microbiome: fact or fiction? Front Cell Infect Microbiol. 2019;9:00148. doi:10.3389/fcimb.2019.00148 PMC6519389 31139578

[8] Shah NB , Allegretti AS , Nigwekar SU , et al. Blood microbiome profile in CKD: a pilot study. Clin J Am Soc Nephrol. 2019;14(5):692‐701. doi:10.2215/CJN.12161018 30962186 PMC6500932

[9] Amar J , Lelouvier B , Servant F , et al. Blood microbiota modification after myocardial infarction depends upon low‐density lipoprotein cholesterol levels. J Am Heart Assoc. 2019;8(19):e011797. doi:10.1161/JAHA.118.011797 31566105 PMC6806051

[10] Lelouvier B , Servant F , Païssé S , et al. Changes in blood microbiota profiles associated with liver fibrosis in obese patients: a pilot analysis. Hepatology. 2016;64(6):2015‐2027. doi:10.1002/hep.28829 27639192

[11] Païssé S , Valle C , Servant F , et al. Comprehensive description of blood microbiome from healthy donors assessed by 16 S targeted metagenomic sequencing. Transfusion. 2016;56(5):1138‐1147. doi:10.1111/trf.13477 26865079

[12] Olde Loohuis LM , Mangul S , Ori AAPSA , et al. Transcriptome analysis in whole blood reveals increased microbial diversity in schizophrenia. Nature. 2018;8(1):1‐9. doi:10.1038/s41398-018-0107-9 PMC5943399 29743478

[13] Glyn T , Purcell RV . Circulating bacterial DNA: a new paradigm for cancer diagnostics. Front Med (Lausanne). 2022;9:643. 10.3389/fmed.2022.831096 PMC9013860 35445046

[14] Salihoğlu R , Önal‐Süzek T . Tissue microbiome associated with human diseases by whole transcriptome sequencing and 16 S metagenomics. Front Genet. 2021;12:585556. 33747035 10.3389/fgene.2021.585556 PMC7970108

[15] Ghaemi F , Fateh A , Sepahy AA , Zangeneh M , Ghanei M , Siadat SD . Blood microbiota composition in Iranian pre‐diabetic and type 2 diabetic patients. Hum Antibodies. 2021;29:243‐248. 34151785 10.3233/HAB-210450

[16] Dereschuk K , Apostol L , Ranjan I , et al. Identification of lung and blood microbiota implicated in COVID‐19 prognosis. Cell. 2021;10(6):1452. 10.3390/cells10061452 PMC8226556 34200572

[17] Chakaroun RM , Massier L , Heintz‐Buschart A , et al. Circulating bacterial signature is linked to metabolic disease and shifts with metabolic alleviation after bariatric surgery. Genome Med. 2021;13(1):1‐21. 34158092 10.1186/s13073-021-00919-6 PMC8218394

[18] Gedgaudas R , Bajaj JS , Skieceviciene J , et al. Circulating microbiome in patients with portal hypertension. Gut Microbes. 2022;14(1):2029674. doi:10.1080/19490976.2022.2029674/SUPPL_FILE/KGMI_A_2029674_SM1876.ZIP 35130114 PMC8824227

[19] Wang Q , Zhao L , Bin Y , et al. Blood bacterial 16 S rRNA gene alterations in women with polycystic ovary syndrome. Front Endocrinol (Lausanne). 2022;13:113. 10.3389/fendo.2022.814520 PMC8908962 35282443

[20] Khan I , Khan I , Kakakhel MA , Xiaowei Z , Ting M . Comparison of microbial populations in the blood of patients with myocardial infarction and healthy individuals. Front Microbiol. 2022;13:1‐13. doi:10.3389/fmicb.2022.845038 PMC9176212 35694288

[21] Khan I , Khan I , Usman M . Analysis of the blood bacterial composition of patients with acute coronary syndrome and chronic coronary syndrome. Front Cell Infect Microbiol. 2022;12:1‐15. doi:10.3389/fcimb.2022.943808 PMC9577097 36268223

[22] Sekirov I , Russell SL , Antunes LCM , Finlay BB . Gut microbiota in health and disease. Physiol Rev. 2010;90(3):859‐904. 20664075 10.1152/physrev.00045.2009

[23] Wang KE , Bennett B , Koeth R , Nature BL . Gut flora metabolism of phosphatidylcholine promotes cardiovascular disease. Nature. 2011;472(7341):57‐63. 21475195 10.1038/nature09922 PMC3086762

[24] Chen K , Zheng XH , Feng M , et al. Gut microbiota‐dependent metabolite trimethylamine N‐oxide contributes to cardiac dysfunction in western diet‐induced obese mice. Front Physiol. 2017;8:139. 28377725 10.3389/fphys.2017.00139 PMC5359299

[25] Rogler G , Rosano G . The heart and the gut. Eur Heart J. 2014;35(7):426‐430. 23864132 10.1093/eurheartj/eht271

[26] Brown JM , Hazen SL . The gut microbial endocrine organ: bacterially derived signals driving cardiometabolic diseases. Annu Rev Med. 2015;66:343‐359. 25587655 10.1146/annurev-med-060513-093205 PMC4456003

[27] Leung C , Rivera L , Furness JB , Angus PW . The role of the gut microbiota in NAFLD. Nat Rev Gastroenterol Hepatol. 2016;13(7):412‐425. 27273168 10.1038/nrgastro.2016.85

[28] Munford RS . Endotoxemia—menace, marker, or mistake? J Leukoc Biol. 2016;100(4):687‐698. 27418356 10.1189/jlb.3RU0316-151R PMC5014740

[29] Ricci V , Carcione D , Messina S , Colombo GI , D'alessandra Y , D'alessandra Y . Circulating 16 s rna in biofluids: extracellular vesicles as mirrors of human microbiome? Int J Mol Sci. 2020;21(23):1‐14. doi:10.3390/ijms21238959 PMC7728300 33255779

[30] Klindworth A , Pruesse E , Schweer T , et al. Evaluation of general 16 S ribosomal RNA gene PCR primers for classical and next‐generation sequencing‐based diversity studies. Nucleic Acids Res. 2013;41(1):e1. 22933715 10.1093/nar/gks808 PMC3592464

[31] Nikkari S , McLaughlin IJ , Bi W , Dodge DE , Relman DA . Does blood of healthy subjects contain bacterial ribosomal DNA? J Clin Microbiol. 2001;39(5):1956‐1959. doi:10.1128/JCM.39.5.1956-1959.2001 11326021 PMC88056

[32] Gallè F , Valeriani F , Cattaruzza MS , et al. Exploring the association between physical activity and gut microbiota composition: a review of current evidence. Ann Ig. 2019;31(6):582‐589. 31616902 10.7416/ai.2019.2318

[33] Hacioglu A , Gundogdu A , Nalbantoglu U , et al. Gut microbiota in patients with newly diagnosed acromegaly: a pilot cross‐sectional study. Pituitary. 2021;24:1‐11. 33721175 10.1007/s11102-021-01137-4

[34] Potgieter M , Bester J , Kell DB , Pretorius E . The dormant blood microbiome in chronic, inflammatory diseases. FEMS Microbiol Rev. 2015;39(4):567‐591. doi:10.1093/femsre/fuv013 25940667 PMC4487407

[35] Bhattacharyya M , Ghosh T , Shankar S , Tomar N . The conserved phylogeny of blood microbiome. Mol Phylogenet Evol. 2017;109:404‐408. 28216014 10.1016/j.ympev.2017.02.001

[36] Rizzatti G , Lopetuso L , Gibiino G . Proteobacteria: a common factor in human diseases. Biomed Res Int. 2017;2017:9351507. https://www.hindawi.com/journals/bmri/2017/9351507/abs/ 29230419 10.1155/2017/9351507 PMC5688358

[37] Gevers D , Knight R , Petrosino JF , et al. The human microbiome project: a community resource for the healthy human microbiome. PLoS Biol. 2012;10(8):e1001377. 22904687 10.1371/journal.pbio.1001377 PMC3419203

[38] Proctor LM , Creasy HH , Fettweis JM , et al. The integrative human microbiome project. Nature. 2019;569(7758):641‐648. 31142853 10.1038/s41586-019-1238-8 PMC6784865

[39] Pasolli E , Asnicar F , Manara S , et al. Extensive unexplored human microbiome diversity revealed by over 150,000 genomes from metagenomes spanning age, geography, and lifestyle. Cell. 2019;176(3):649‐662. 30661755 10.1016/j.cell.2019.01.001 PMC6349461

[40] Dinakaran V , Rathinavel A , Pushpanathan M , Sivakumar R , Gunasekaran P , Rajendhran J . Elevated levels of circulating DNA in cardiovascular disease patients: metagenomic profiling of microbiome in the circulation. PLoS One. 2014;9(8):e105221. doi:10.1371/journal.pone.0105221 25133738 PMC4136842

[41] Rajendhran J , Shankar M , Dinakaran V , Rathinavel A , Gunasekaran P . Contrasting circulating microbiome in cardiovascular disease patients and healthy individuals. Int J Cardiol. 2013;168(5):5118‐5120. doi:10.1016/j.ijcard.2013.07.232 23962776

[42] Dinakaran V , John L , Rathinavel A , Gunasekaran P , Rajendhran J . Prevalence of bacteria in the circulation of cardiovascular disease patients, Madurai, India. Heart Lung Circ. 2012;21(5):281‐283. doi:10.1016/j.hlc.2012.02.007 22459237

[43] Zhou X , Li Y , Guo J , et al. Gut‐dependent microbial translocation induces inflammation and cardiovascular events after ST‐elevation myocardial infarction. Microbiome. 2018;6(1):1‐17. doi:10.1186/s40168-018-0441-4 29615110 PMC5883284

[44] Fong PC , Boss DS , Yap TA , et al. Inhibition of poly (ADP‐ribose) polymerase in tumors from BRCA mutation carriers. N Engl J Med. 2009;361(2):123‐134. 19553641 10.1056/NEJMoa0900212

[45] Cowan LT , Lutsey PL , Pankow JS , Matsushita K , Ishigami J , Lakshminarayan K . Inpatient and outpatient infection as a trigger of cardiovascular disease: the ARIC study. J Am Heart Assoc. 2018;7(22):e009683. doi:10.1161/JAHA.118.009683 30571501 PMC6404437

[46] Priyamvara A , Dey AK , Bandyopadhyay D , et al. Periodontal inflammation and the risk of cardiovascular disease. Curr Atheroscler Rep. 2020;22(7):28. doi:10.1007/s11883-020-00848-6.pdf 32514778

[47] Zhou X , Li Y , Guo J , et al. Gut‐dependent microbial translocation induces inflammation and cardiovascular events after ST‐elevation myocardial infarction. Microbiome. 2018;6(1):66. doi:10.1186/s40168-018-0441-4 29615110 PMC5883284

[48] Amar J , Lange C , Payros G , et al. Blood microbiota dysbiosis is associated with the onset of cardiovascular events in a large general population: the DESIR study. PLoS One. 2013;8(1):e54461. doi:10.1371/journal.pone.0054461 23372728 PMC3555817

[49] Velmurugan G , Dinakaran V , Rajendhran J , Swaminathan K . Blood microbiota and circulating microbial metabolites in diabetes and cardiovascular disease. Trends Endocrinol Metab. 2020;31(11):835‐847. doi:10.1016/j.tem.2020.01.013 33086076

[50] Dominy SS , Lynch C , Ermini F , et al. Porphyromonas gingivalis in Alzheimer's disease brains: evidence for disease causation and treatment with small‐molecule inhibitors. Sci Adv. 2019;5(1):eaau3333. 30746447 10.1126/sciadv.aau3333 PMC6357742

[51] Kell DB , Pretorius E . On the translocation of bacteria and their lipopolysaccharides between blood and peripheral locations in chronic, inflammatory diseases: the central roles of LPS and LPS‐induced cell death. Integr Biol. 2015;7(11):1339‐1377. 10.1039/c5ib00158g 26345428

[52] De Waal GM , Engelbrecht L , Davis T , De Villiers WJS , Kell DB , Pretorius E . Correlative light‐electron microscopy detects lipopolysaccharide and its association with fibrin fibres in Parkinson's disease, alzheimer's disease and type 2 diabetes mellitus. Sci Rep. 2018;8(1):1‐12. 30429533 10.1038/s41598-018-35009-y PMC6235901

[53] Hammad DBM , Hider SL , Liyanapathirana VC , Tonge DP . Molecular characterization of circulating microbiome signatures in rheumatoid arthritis. Front Cell Infect Microbiol. 2020;9:9. doi:10.3389/fcimb.2019.00440 PMC6987042 32039040

[54] Sumida K , Pierre JF , Han Z , et al. Circulating microbial signatures and cardiovascular death in patients with&nbsp;ESRD. Kidney Int Rep. 2021;6:2617‐2628. doi:10.1016/j.ekir.2021.07.023 34622101 PMC8484116

[55] Serrano‐Villar S , Sanchez‐Carrillo S , Talavera‐Rodríguez A , et al. Blood bacterial profiles associated with human immunodeficiency virus infection and immune recovery. J Infect Dis. 2021;223(3):471‐481. 32601702 10.1093/infdis/jiaa379

[56] Joura M , Brunner A , Nemes‐Nikodém É , et al. Interactions between immune system and the microbiome of skin, blood and gut in pathogenesis of rosacea. Acta Microbiol Immunol Hung. 2021;68(1):1‐6. doi:10.1556/030.2021.01366.xml 33522984

[57] Lee EJ , Sung J , Kim HL , Kim HN . Whole‐genome sequencing reveals age‐specific changes in the human blood microbiota. J Pers Med. 2022;12(6):939. 35743724 10.3390/jpm12060939 PMC9225573

[58] Lee HJ , Seo HI , Cha HY , Yang YJ , Kwon SH , Yang SJ . Diabetes and Alzheimer's disease: mechanisms and nutritional aspects. Clin Nutr Res. 2018;7(4):229‐240. doi:10.7762/cnr.2018.7.4.229 30406052 PMC6209735

[59] Spanogiannopoulos P , Bess EN , Carmody RN , Turnbaugh PJ . The microbial pharmacists within us: a metagenomic view of xenobiotic metabolism. Nat Rev Microbiol. 2016;14(5):273‐287. 26972811 10.1038/nrmicro.2016.17 PMC5243131

[60] Velmurugan G , Ramprasath T , Swaminathan K , et al. Gut microbial degradation of organophosphate insecticides‐induces glucose intolerance via gluconeogenesis. Genome Biol. 2017;18(1):1‐18. 28115022 10.1186/s13059-016-1134-6 PMC5260025

[61] Velmurugan G . Gut microbiota in toxicological risk assessment of drugs and chemicals: the need of hour. Gut Microbes. 2018;9(5):465‐468. 29509057 10.1080/19490976.2018.1445955 PMC6219644

[62] Koeth RA , Wang Z , Levison BS , Hazen SL . Intestinal microbiota metabolism of l‐carnitine, a nutrient in red meat, promotes atherosclerosis. Nat Med. 2013;19(5):576‐585. 23563705 10.1038/nm.3145 PMC3650111

[63] Wang Z , Tang WHW , Buffa JA , et al. Prognostic value of choline and betaine depends on intestinal microbiota‐generated metabolite trimethylamine‐N‐oxide. Eur Heart J. 2014;35(14):904‐910. 24497336 10.1093/eurheartj/ehu002 PMC3977137

[64] Tang WHW , Wang Z , Kennedy DJ , et al. Gut microbiota‐dependent trimethylamine N‐oxide (TMAO) pathway contributes to both development of renal insufficiency and mortality risk in chronic kidney disease. Circ Res. 2015;116(3):448‐455. 25599331 10.1161/CIRCRESAHA.116.305360 PMC4312512

[65] Li XS , Obeid S , Klingenberg R , et al. Gut microbiota‐dependent trimethylamine N‐oxide in acute coronary syndromes: a prognostic marker for incident cardiovascular events beyond traditional risk factors. Eur Heart J. 2017;38(11):814‐824. 28077467 10.1093/eurheartj/ehw582 PMC5837488

[66] Tang WHW , Wang Z , Fan Y , et al. Prognostic value of elevated levels of intestinal microbe‐generated metabolite trimethylamine‐N‐oxide in patients with heart failure: refining the gut hypothesis. J Am Coll Cardiol. 2014;64(18):1908‐1914. 25444145 10.1016/j.jacc.2014.02.617 PMC4254529

[67] Senthong V , Wang Z , Li XS , et al. Intestinal microbiota‐generated metabolite trimethylamine‐N‐oxide and 5‐year mortality risk in stable coronary artery disease: the contributory role of intestinal microbiota in a COURAGE‐like patient cohort. J Am Heart Assoc. 2016;5(6):e002816. 27287696 10.1161/JAHA.115.002816 PMC4937244

[68] Shi K , Wang F , Jiang H , et al. Gut bacterial translocation may aggravate microinflammation in hemodialysis patients. Dig Dis Sci. 2014;59(9):2109‐2117. 24828917 10.1007/s10620-014-3202-7

[69] Tang WHW , Wang Z , Levison BS , et al. Intestinal microbial metabolism of phosphatidylcholine and cardiovascular risk. N Engl J Med. 2013;368(17):1575‐1584. doi:10.1056/nejmoa1109400 23614584 PMC3701945

[70] Brunt VE , Gioscia‐Ryan RA , Casso AG , et al. Trimethylamine‐N‐oxide promotes age‐related vascular oxidative stress and endothelial dysfunction in mice and healthy humans. Hypertension. 2020;76(1):101‐112. 32520619 10.1161/HYPERTENSIONAHA.120.14759 PMC7295014

[71] Chen S , Henderson A , Petriello MC , et al. Trimethylamine N‐oxide binds and activates PERK to promote metabolic dysfunction. Cell Metab. 2019;30(6):1141‐1151. 31543404 10.1016/j.cmet.2019.08.021

[72] Wang Z , Roberts AB , Buffa JA , et al. Non‐lethal inhibition of gut microbial trimethylamine production for the treatment of atherosclerosis. Cell. 2015;163(7):1585‐1595. 26687352 10.1016/j.cell.2015.11.055 PMC4871610

[73] Roberts AB , Gu X , Buffa JA , et al. Development of a gut microbe–targeted nonlethal therapeutic to inhibit thrombosis potential. Nat Med. 2018;24(9):1407‐1417. 30082863 10.1038/s41591-018-0128-1 PMC6129214

[74] Ahmad AF , Dwivedi G , O'Gara F , Caparros‐Martin J , Ward NC . The gut microbiome and cardiovascular disease: current knowledge and clinical potential. Am J Physiol Circ Physiol. 2019;317(5):H923‐H938. 10.1152/ajpheart.00376.2019 31469291

[75] Blacher E , Levy M , Tatirovsky E , Elinav E . Microbiome‐modulated metabolites at the interface of host immunity. J Immunol. 2017;198(2):572‐580. 28069752 10.4049/jimmunol.1601247

[76] Donohoe DR , Garge N , Zhang X , et al. The microbiome and butyrate regulate energy metabolism and autophagy in the mammalian colon. Cell Metab. 2011;13(5):517‐526. 21531334 10.1016/j.cmet.2011.02.018 PMC3099420

[77] Pluznick JL , Protzko RJ , Gevorgyan H , et al. Olfactory receptor responding to gut microbiota‐derived signals plays a role in renin secretion and blood pressure regulation. Proc Natl Acad Sci. 2013;110(11):4410‐4415. 23401498 10.1073/pnas.1215927110 PMC3600440

[78] Wang L , Zhu Q , Lu A , et al. Sodium butyrate suppresses angiotensin II‐induced hypertension by inhibition of renal (pro) renin receptor and intrarenal renin–angiotensin system. J Hypertens. 2017;35(9):1899‐1908. 28509726 10.1097/HJH.0000000000001378 PMC11157961

[79] Natarajan N , Hori D , Flavahan S , et al. Gut microbiota in health and disease: microbial short chain fatty acid metabolites lower blood pressure via endothelial G protein‐coupled receptor 41. Physiol Genomics. 2016;48(11):826‐834. 27664183 10.1152/physiolgenomics.00089.2016 PMC6223570

[80] Pluznick J . A novel SCFA receptor, the microbiota, and blood pressure regulation. Gut Microbes. 2014;5(2):202‐207. 24429443 10.4161/gmic.27492 PMC4063845

[81] Bartolomaeus H , Balogh A , Yakoub M , et al. Short‐chain fatty acid propionate protects from hypertensive cardiovascular damage. Circulation. 2019;139(11):1407‐1421. 30586752 10.1161/CIRCULATIONAHA.118.036652 PMC6416008

[82] Molinaro A , Marschall HU . Bile acid metabolism and FXR‐mediated effects in human cholestatic liver disorders. Biochem Soc Trans. 2022;50(1):361‐373. 35191955 10.1042/BST20210658

[83] Raedsch R , Lauterburg BH , Hofmann AF . Altered bile acid metabolism in primary biliary cirrhosis. Dig Dis Sci. 1981;26(5):394‐401. 7249880 10.1007/BF01313580

[84] Fiorucci S , Distrutti E . The pharmacology of bile acids and their receptors. Handb Exp Pharmacol. 2019;256:3‐18. 31201555 10.1007/164_2019_238

[85] Doden H , Sallam LA , Devendran S , et al. Metabolism of oxo‐bile acids and characterization of recombinant 12α‐hydroxysteroid dehydrogenases from bile acid 7α‐dehydroxylating human gut bacteria. Appl Environ Microbiol. 2018;84(10):e00235‐18. 29549099 10.1128/AEM.00235-18 PMC5930368

[86] Kahlon TS , Chiu MCM , Chapman MH . Steam cooking significantly improves in vitro bile acid binding of collard greens, kale, mustard greens, broccoli, green bell pepper, and cabbage. Nutr Res. 2008;28(6):351‐357. 19083431 10.1016/j.nutres.2008.03.007

[87] Rainer PP , Primessnig U , Harenkamp S , et al. Bile acids induce arrhythmias in human atrial myocardium—implications for altered serum bile acid composition in patients with atrial fibrillation. Heart. 2013;99(22):1685‐1692. 23894089 10.1136/heartjnl-2013-304163

[88] Gutierrez A , Ratliff EP , Andres AM , Huang X , McKeehan WL , Davis RA . Bile acids decrease hepatic paraoxonase 1 expression and plasma high‐density lipoprotein levels via FXR‐mediated signaling of FGFR4. Arterioscler Thromb Vasc Biol. 2006;26(2):301‐306. 16284190 10.1161/01.ATV.0000195793.73118.b4

[89] Sun L , Xie C , Wang G , et al. Gut microbiota and intestinal FXR mediate the clinical benefits of metformin. Nat Med. 2018;24(12):1919‐1929. 30397356 10.1038/s41591-018-0222-4 PMC6479226

[90] Hayashi T , Yamashita T , Watanabe H , et al. Gut microbiome and plasma microbiome‐related metabolites in patients with decompensated and compensated heart failure. Circ J. 2018;83(1):182‐192. 30487369 10.1253/circj.CJ-18-0468

[91] Brial F , Le Lay A , Dumas MEE , Gauguier D . Implication of gut microbiota metabolites in cardiovascular and metabolic diseases. Cell Mol Life Sci. 2018;75(21):3977‐3990. doi:10.1007/s00018-018-2901-1 30101405 PMC6182343

[92] Cason CA , Dolan KT , Sharma G , et al. Plasma microbiome‐modulated indole‐and phenyl‐derived metabolites associate with advanced atherosclerosis and postoperative outcomes. J Vasc Surg. 2018;68(5):1552‐1562. 29248242 10.1016/j.jvs.2017.09.029 PMC5999545

[93] Lam V , Su J , Hsu A , Gross GJ , Salzman NH , Baker JE . Intestinal microbial metabolites are linked to severity of myocardial infarction in rats. PLoS One. 2016;11(8):e0160840. 27505423 10.1371/journal.pone.0160840 PMC4978455

[94] Poesen R , Claes K , Evenepoel P , et al. Microbiota‐derived phenylacetylglutamine associates with overall mortality and cardiovascular disease in patients with CKD. J Am Soc Nephrol. 2016;27(11):3479‐3487. 27230658 10.1681/ASN.2015121302 PMC5084895

[95] Nemet I , Saha PP , Gupta N , et al. A cardiovascular disease‐linked gut microbial metabolite acts via adrenergic receptors. Cell. 2020;180(5):862‐877. 32142679 10.1016/j.cell.2020.02.016 PMC7402401

[96] Chen L , Ishigami T , Nakashima‐Sasaki R , et al. Commensal microbe‐specific activation of B2 cell subsets contributes to atherosclerosis development independently of lipid metabolism. EBioMedicine. 2016;13:237‐247. 27810309 10.1016/j.ebiom.2016.10.030 PMC5264349

[97] Suez J , Korem T , Zeevi D , et al. Artificial sweeteners induce glucose intolerance by altering the gut microbiota. Nature. 2014;514:181‐186. doi:10.1038/nature13793 25231862

[98] Martin F , Wang Y , Sprenger N , et al. Probiotic modulation of symbiotic gut microbial–host metabolic interactions in a humanized microbiome mouse model. Mol Syst Biol. 2008;4:4100190. doi:10.1038/msb4100190 PMC2238715 18197175

[99] Brown J , Hazen SL , Chemistry SHJ of biological . Targeting of microbe‐derived metabolites to improve human health: the next frontier for drug discovery. J Biol Chem. 2017;292(21):8560‐8568. 28389555 10.1074/jbc.R116.765388 PMC5448085

[100] Cully M . Microbiome therapeutics go small molecule. Nat Rev Drug Discov. 2019;18(8):569‐573. 31367062 10.1038/d41573-019-00122-8

[101] Hornung BVH , Zwittink RD , Kuijper EJ . Issues and current standards of controls in microbiome research. FEMS Microbiol Ecol. 2019;95(5):fiz045. 30997495 10.1093/femsec/fiz045 PMC6469980

[102] Eisenhofer R , Minich JJ , Marotz C , Cooper A , Knight R , Weyrich LS . Contamination in low microbial biomass microbiome studies: issues and recommendations. Trends Microbiol. 2019;27(2):105‐117. 30497919 10.1016/j.tim.2018.11.003

[103] Turner P , Turner C , Jankhot A , et al. A longitudinal study of Streptococcus pneumoniae carriage in a cohort of infants and their mothers on the Thailand‐Myanmar border. PLoS One. 2012;7(5):e38271. 22693610 10.1371/journal.pone.0038271 PMC3365031

[104] Salter SJ , Cox MJ , Turek EM , et al. Reagent and laboratory contamination can critically impact sequence‐based microbiome analyses. BMC Biol. 2014;12(1):1‐12. 25387460 10.1186/s12915-014-0087-z PMC4228153

